# Interprofessional and interdisciplinary simulation-based training leads to safe sedation procedures in the emergency department

**DOI:** 10.1186/s13049-016-0291-7

**Published:** 2016-08-02

**Authors:** Thomas C. Sauter, Wolf E. Hautz, Simone Hostettler, Monika Brodmann-Maeder, Luca Martinolli, Beat Lehmann, Aristomenis K. Exadaktylos, Dominik G. Haider

**Affiliations:** Department of Emergency Medicine, Inselspital, University Hospital Bern, Freiburgstrasse, 3010 Bern, Switzerland

**Keywords:** Conscious sedation, Interprofessional education, Emergency department

## Abstract

**Background:**

Sedation is a procedure required for many interventions in the Emergency department (ED) such as reductions, surgical procedures or cardioversions. However, especially under emergency conditions with high risk patients and rapidly changing interdisciplinary and interprofessional teams, the procedure caries important risks. It is thus vital but difficult to implement a standard operating procedure for sedation procedures in any ED. Reports on both, implementation strategies as well as their success are currently lacking. This study describes the development, implementation and clinical evaluation of an interprofessional and interdisciplinary simulation-based sedation training concept.

**Methods:**

All physicians and nurses with specialised training in emergency medicine at the Berne University Department of Emergency Medicine participated in a mandatory interdisciplinary and interprofessional simulation-based sedation training. The curriculum consisted of an individual self-learning module, an airway skill training course, three simulation-based team training cases, and a final practical learning course in the operating theatre. Before and after each training session, self-efficacy, awareness of emergency procedures, knowledge of sedation medication and crisis resource management were assessed with a questionnaire. Changes in these measures were compared via paired tests, separately for groups formed based on experience and profession. To assess the clinical effect of training, we collected patient and team satisfaction as well as duration and complications for all sedations in the ED within the year after implementation. We further compared time to beginning of procedure, time for duration of procedure and time until discharge after implementation with the one year period before the implementation. Cohen’s d was calculated as effect size for all statistically significant tests.

**Results:**

Fifty staff members (26 nurses and 24 physicians) participated in the training. In all subgroups, there is a significant increase in self-efficacy and knowledge with high effect size (*d*_*z*_ = 1.8). The learning is independent of profession and experience level. In the clinical evaluation after implementation, we found no major complications among the sedations performed. Time to procedure significantly improved after the introduction of the training (*d* = 0.88).

**Discussion:**

Learning is independent of previous working experience and equally effective in raising the self-efficacy and knowledge in all professional groups. Clinical outcome evaluation confirms the concepts safety and feasibility.

**Conclusion:**

An interprofessional and interdisciplinary simulation-based sedation training is an efficient way to implement a conscious sedation concept in an ED.

## Background

In any emergency department (ED), sedation is a necessary practice to facilitate urgent medical interventions (e.g., cardioversion, repositioning fractures or other painful procedures).

Common risks of sedation — not only in the ED — include aspiration, hypotension, hypoventilation and impaired reflexes, causing adverse effects [1–5]. Furthermore, the need for sedation does not arise very often which limits the personal experience of ED staff with the procedure. As a consequence, ED staff might have low levels of confidence in sedation.

In contrast to the UK or North America, structured training for emergency physicians is lacking in many European countries, including Switzerland. Moreover, the ED staff in many countries consist of physicians from a variety of specialities (e.g., internal medicine, orthopaedics or general surgery). Consequently, sedation is usually provided either by anaesthetists or by emergency physicians who then often follow non-standardised procedures in accordance with their personal experience.

Anaesthetists (working in the unfamiliar environment of the ED) and ED physicians (using unfamiliar drugs and sedation protocols) may alienate ED staff. These factors (e.g., uncertainty among team members, rarely performed procedures in unfamiliar settings, the lack of standardisation, disparate perceptions and expectations of different team members) are commonly referred to as “human factors” and are a major cause of medical errors [6]. The importance of these human factors and team training was emphasised in the 2010 Helsinki Declaration on Patient Safety in Anaesthesiology, which identified education as a key strategy to improve patient safety [7].

In emergency teams, as well as in other teams, there is growing evidence that the implementation of crisis resource principles improves teamwork, reduces medical errors and ultimately improves patient outcome. One approach to implementation — borrowed from aviation — has traditionally been the use of check-lists and standard operating procedures, which have been shown to dramatically reduce medical error in the operating theatre [8, 9]. Furthermore, inter-professional education has been stressed as an important option to reduce medical error [10].

Because of the necessity to perform team-based tasks such as procedural sedation, the ED is predestined for the implementation of interprofessional education. The importance of such implementation is reflected in a “Call to action for emergency medicine” by Wilbur et al. [11].

However, the literature describes few specific training programs for sedation procedures in the ED, which leaves educators in a difficult position. Two recent reports have demonstrated the feasibility of simulation-based training in sedation for ED teams [12, 13]. However, neither of these conducted an interprofessional training session, the number of participants was low and no clinical outcome evaluation was included.

Therefore we addressed the following research questions:

- Firstly, how can an interprofessional and interdisciplinary sedation training be designed that addresses the needs of the different professions, disciplines and levels of experience of the ED personnel?
- Secondly, what are the clinical effects of the introduction of such a training?

## Methods

All residents, consultant physicians and nurses with special training in emergency medicine in our department mandatorily participated in our sedation training. Our emergency department is a university major trauma centre with about 42,000 emergency admissions per year.

**Fig. 1:**
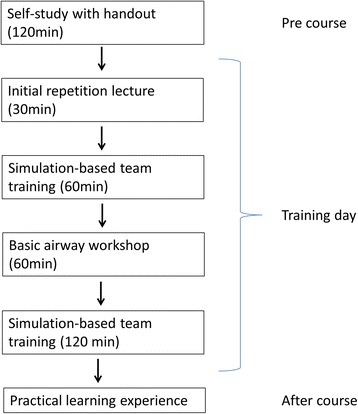
Training schedule

Our training program is structured into several steps (Fig. 1), that are based on Smith’s principles of patient safety. He described three core components of patient safety that should be included in all medical educational programs: guiding principles, body of knowledge and set of tools [7, 14].

### Structure and content of the training course

The first step of the education concept is independent study of a hand-out that has to be completed before the simulation, providing theoretical information about sedation, the ED sedation concept and underlying guidelines. The aim of this step is to familiarise participants with the general concept and to ensure a baseline knowledge about the topic. With regards to content, our concept is based on the guidelines of the American College of Emergency Physicians Clinical Policies Subcommittee [15]. Because the discussion about the best medication used for sedation procedures in the ED (ketamine, midazolam, propofol, fentanyl) is still on-going [16–18], we deliberately did not dictate any specific medication, but demonstrated the advantages and disadvantages of all available medication and provide a standardized framework for the safe conduction of sedation, regardless of the specific medication used.

The training day, as a second step, starts with a short theoretical repetition of the concept and focuses on questions of the participants. This step was introduced to provide participants with the opportunity to clarify any questions that might have arisen during step 1 and to lower the threshold to engage in the simulation activities of step 3.

The third step consists of three simulated scenarios with supervision, interposed with airway skills training. This step is conducted to practice the concepts introduced during the first two steps and to provide participants with the opportunity to train in sedation as well as to increase their familiarity with complications that might arise.

The case scenarios were practiced in real work surroundings with an interprofessional real life team consisting of physicians and nurses. All simulation cases are practiced with a computer-enhanced mannequin simulator (SimMan 3G, Laerdal Medical, Norway). According to our concept, a sedation team consists of at least two trained staff members, including at least one physician. After each case scenario, medical issues as well as human factors were debriefed using video-assistance by an interprofessional (nurse/physician) and interdisciplinary (emergency medicine/anaesthesia) instructor team. Each instructor (6 in total) had a minimum of 1 year of experience in simulation/debriefing and was trained in a simulation instructor course of several days duration. One supervising instructor was present at all training courses in order to ensure continuity in teaching techniques, as well as in the medical contents.

A special focus of our simulation scenarios was on the prevention of possible complications and on emergency procedures. We emphasised the importance and implementation of capnography, as recommended in procedural sedation guidelines and research [19, 20]. The training session included the use of a structured pre-sedation checklist (available on request), including a team timeout, as well as the development and documentation of emergency strategies.

In the airway skill training, we formed small groups of 2–3 participants. Each group was trained using an Adult Airway Management Trainer Manikin (Laerdal, Norway). The airway skills course focussed on bag-valve ventilation techniques (single and two-handed approach), with simple tools such as nasopharyngeal and oropharyngeal airway devices, including insertion techniques, indications and pitfalls. As a rescue tool, the insertion of an laryngeal mask was taught. We did not teach orotracheal intubation in order to lower participants’ anxiety and - most importantly - because the necessary routine to master orotracheal intubation safely would be very difficult to ensure and maintain in our ED setting. All airway tools introduced during training, as well as the necessary medication, are now provided in the ED in a special sedation procedure box and all participants have been familiarised with the equipment provided in the ED through the training.

In the simulation based team training courses, we used three standardised scenarios (available upon request). In each scenario, the focus is on a special medical topic frequently encountered in our ED (e.g., luxated shoulder), as well as on crisis resource management (CRM). For crisis resource management, we used the CRM principles as conceptual framework, as outlined by Gaba and Rall [21–24]. Regarding medical factors, we focused on the main complications that arise in sedation procedures (apnoea, circulatory problems and potentially obstructed airways).

Before and after the simulation-based training course, we used a custom questionnaire to assess confidence and familiarity with the sedation concept, awareness of emergency procedures, knowledge of the sedation medication and knowledge of CRM principles. The acceptance of the specified statements had to be indicated on a 11-point Likert scale ranging from 0 (completely disagree) to 10 (completely agree). All measurement instruments are available upon request.

As the last and fourth step, the simulation-based training day was followed by a separate individual practical learning experience in the department of anaesthesia, where participants further practiced bag-ventilation and the use of the simple airway tools they were familiarized with during training.

Because of the suspected infrequency of sedation procedures and subsequent possible lack of regular experience, we conduct monthly refresher trainings of 3 h duration for all trained staff.

### Implementation and clinical outcome

Starting with the roll-out of the program, it is now mandatory to conduct procedural sedation in our ED in accordance to the trained standards. Every sedation procedure is under on-going evaluation through a questionnaire (available upon request) and the documentation in the patients chart. General factors (type of intervention, administered medication, timeline of the intervention and ER visit as well as reported adverse events) are routinely recorded in the patient chart. Overall satisfaction of the patient, as well as pain experienced during the procedure are monitored. The perceived usefulness of the sedation training for the actual sedation team is further assessed. Additionally, we screened the charts of all patients who underwent a sedation retrospectively for adverse events. To evaluate the usefulness of the sedation program for ED workflow, we compared age, gender, the American Association of Anaesthesiologists - scale (ASA), time to procedure (defined as time until procedure was begun), time for procedure (defined as duration of procedure) and time to discharge in patients sedated for the reduction of a luxated shoulder by the trained ED team in 2015 with the patients treated by anaesthesia teams in 2014.

The study protocol was assessed by the Ethics Committee of the Canton Bern, Switzerland (Req-2016-00134) and was classified as quality control investigation. Therefore no informed patient consent is necessary according to Swiss law.

### Analysis

We compared participants by age group, years of working experience, gender and profession by means of *t*-test (age, experience) and Fishers exact test (gender) as appropriate. Self-assessed knowledge and confidence before and after the training were compared using paired samples Student’s t tests. Time to procedure, time for procedure and time to discharge between patients sedated by the ED team and those cared for by teams of the Department of Anaesthesia were compared by means of Mann–Whitney-U test for unrelated samples, their ASA-classification by means of a univariate ANOVA. All calculations were performed in SPSS Statistics 21 (IBM Coorp.). A p-value of less than 0.05 was considered statistically significant. We calculated Cohen’s *d* as effect size for all statistically significant tests in unrelated samples and *d*_*z*_ for related samples.

## Results

By March 1st 2015, we had included 50 staff-participants in the present study. All participants answered the questionnaire provided. Detailed demographic characteristics are shown in Table 1. Only two physicians and none of the nurses had previous working experience in anaesthesia (4 % of the participants). The number of doctors and nurses were comparable (26 nurses; 52 % vs. 24 physician; 48 %). The largest groups had worked for 6–10 years (23 participants; 46 %) or 11–20 years (16 participants; 32 %).

**Table 1:** Demographic characteristics of all participants with complete questionnaires, *n* = 50

Parameter	Number and % of participants
Age group	
21-30 years	5 (10 %)
31-40 years	33 (66 %)
41-50 years	8 (16 %)
51-60 years	4 (8 %)
Gender (male, female, unknown)	8 (16 %), 34 (68 %), 8 (16 %)
Profession	
Nurse	26 (52 %)
Physician	24 (48 %)
Previous anaesthesia training	2 (4 %)
Years of working experience	
0-5 years	6 (12 %)
6-10 years	23 (46 %)
11-20 years	16 (32 %)
>20 years	5 (10 %)

### Self-efficacy

**Fig. 2:**
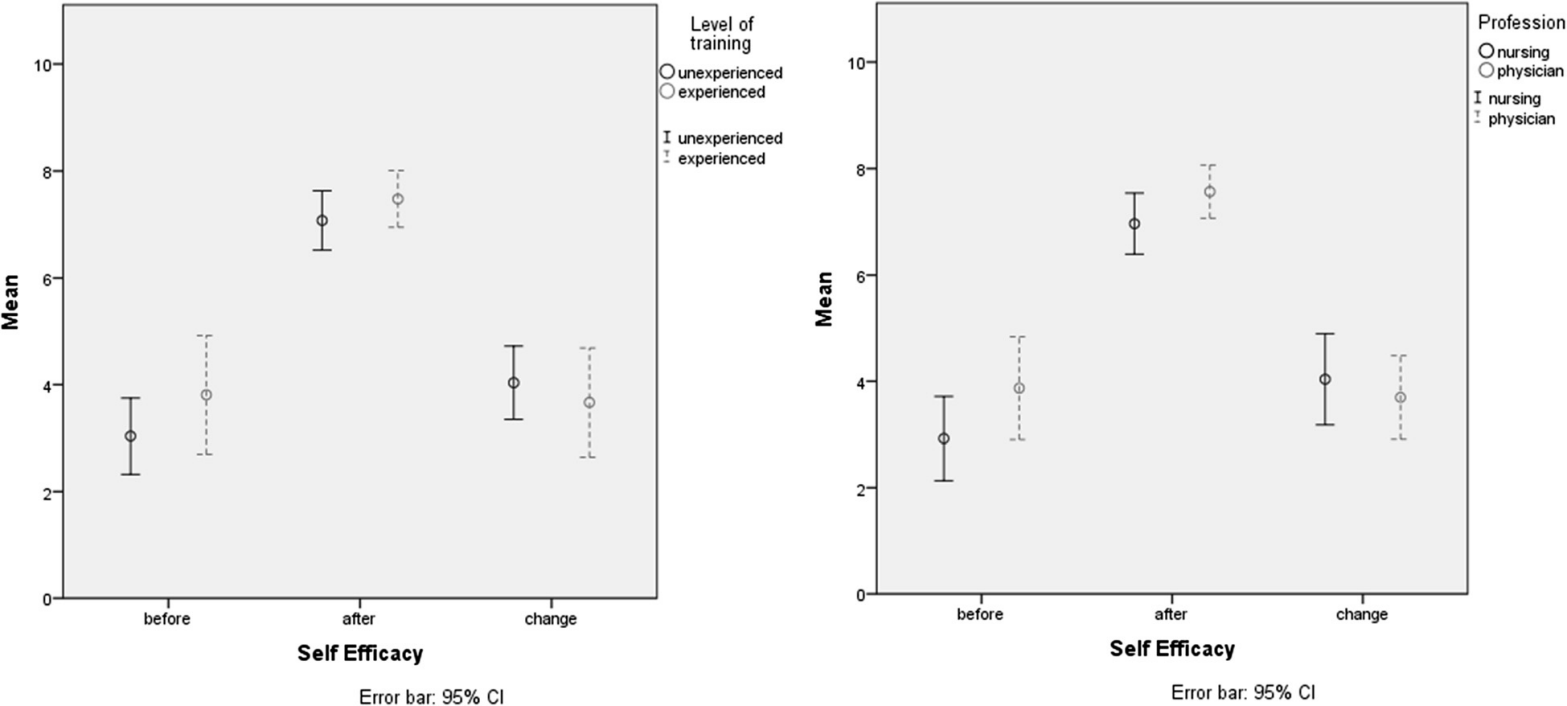
Training effect in self-efficacy depended on level of training (left) and profession (right). Measured on 11-point Likert scales. *n* = 50, *d = 3.98*

We found a highly significant increase in self-efficacy from before to after training in all participants, as well as in all subgroups (all *p* < 0.01, *d*_*z*_ for all participants = 1.82, Table 2). A subgroup analysis found no significant difference in the training effect between experienced and inexperienced participants (*p* = 0.745), or between physicians and nurses (*p* = 0.36). The training effect depended on the level of training and profession and is shown in Fig. 2.

**Table 2:** Comparison of answers to questionnaire before and after training; *n* = 50, mean (standard deviation), (Student’s *t* test for paired samples, all *p* < 0.01)

Parameter	Before	After	Change Δ
Confidence			
Cumulative	3.3 (±2.1)	7.2 (±1.3)	3.8 (±2.1)
Physicians; nurses	2.9 (±2.0); 3.8 (±2.3)	7.6 (±1.2); 7.0 (±1.4)	4.0 (±2.1); 3.5 (±2.0)
Inexperienced; experienced	3.0 (±1.8); 3.8 (±2.4)	7.1 (±1.4); 7.5 (±1.2)	3.9 (±2.0); 3.7 (±2.2)
Emergencies			
Cumulative	4.2 (±2.1)	7.6 (±1.5)	3.4 (±2.0)
Physicians/nurses	4.8 (±2.2); 3.6 (±2.0)	7.7 (±1.6); 7.5 (±1.4)	2.9 (±2.0); 3.9 (±2.0)
Inexperienced; experienced	4.6 (±2.2); 3.7 (±2.0)	7.7 (±1.3); 7.4 (±1.8)	3.1 (±2.1); 3.7 (±2.0)
Medication			
Cumulative	3.6 (±1.8)	6.9 (±1.5)	3.2 (±1.7)
Physicians/nurses	4.3 (±1.7); 3.0 (±1.6)	7.4 (±1.2); 6.4 (±1.6)	3.1 (±1.7); 3.4 (±1.7)
Inexperienced; experienced	3.7 (±1.9); 3.7 (±1.5)	6.8 (±1.7); 7.0 (±1.1)	3.1 (±1.7); 3.3 (±1.7)
CRM-principles			
Cumulative	2.2 (±2.9)	6.6 (±2.4)	4.5 (±3.0)
Physicians/nurses	2.9 (±3.4); 1.3 (±2.1)	7.2 (±2.2); 6.0 (±2.5)	4.3 (±3.2); 4.7 (±2.9)
Inexperienced; experienced	1.9 (±2.9); 2.5 (±2.9)	6.8 (±2.5); 6.3 (±2.1)	4.9 (±3.2); 3.8 (2.6)

### Emergency situational and medical knowledge

We found that emergency situational knowledge in self-assessment as well as knowledge of medication for sedation significantly increased from before to after the training course, both overall and in groups by experience and profession (all *p* < 0.01, *d*_*z*_ for all participants = 1.65 respectively *d*_*z*_ for all participants = 1.89, Table 2).

### CRM-principles and guidelines

Twenty-one participants (42 %) indicated that they had not used CRM-guidelines at all before the training course. After the training courses, participants were asked specifically about the CRM principles they used during the training and were planning to use regularly. The most frequent free text answers were “Call for help early” and “Communicate effectively; Closed loop communication”. As with the previous questions, there was a significant increase in self-assessed knowledge in all subgroups (all *p* < 0.01, *d*_*z*_ for all participants = 1.49, Table 2) without significant difference in subgroups for experience (*p* = 0.257) and profession (*p* = 0.703).

### Clinical outcome evaluation

In 2015, the department of emergency medicine conducted a total of 43 sedations of which 38 are fully documented. The average time to the procedure requiring sedation was 144 min (SD = 146 min), the average time to discharge was 387 min (SD = 255 min). Of a total of 43 patients sedated in the ED in 2015, four patients encountered minor complications that could be managed by the ED team: one patient required an oropharyngeal airway, one patient reported hallucinations, one patient remembered minor pain during the procedure and one patient vomited after a sedation. We did not encounter complications requiring the intervention of an anaesthetist, advanced airway management or hospitalization of the patient. All patients reported, that they would again agree to the form of sedation chosen. All staff reported that the simulation training described above was helpful to them for the current procedure.

**Fig. 3:**
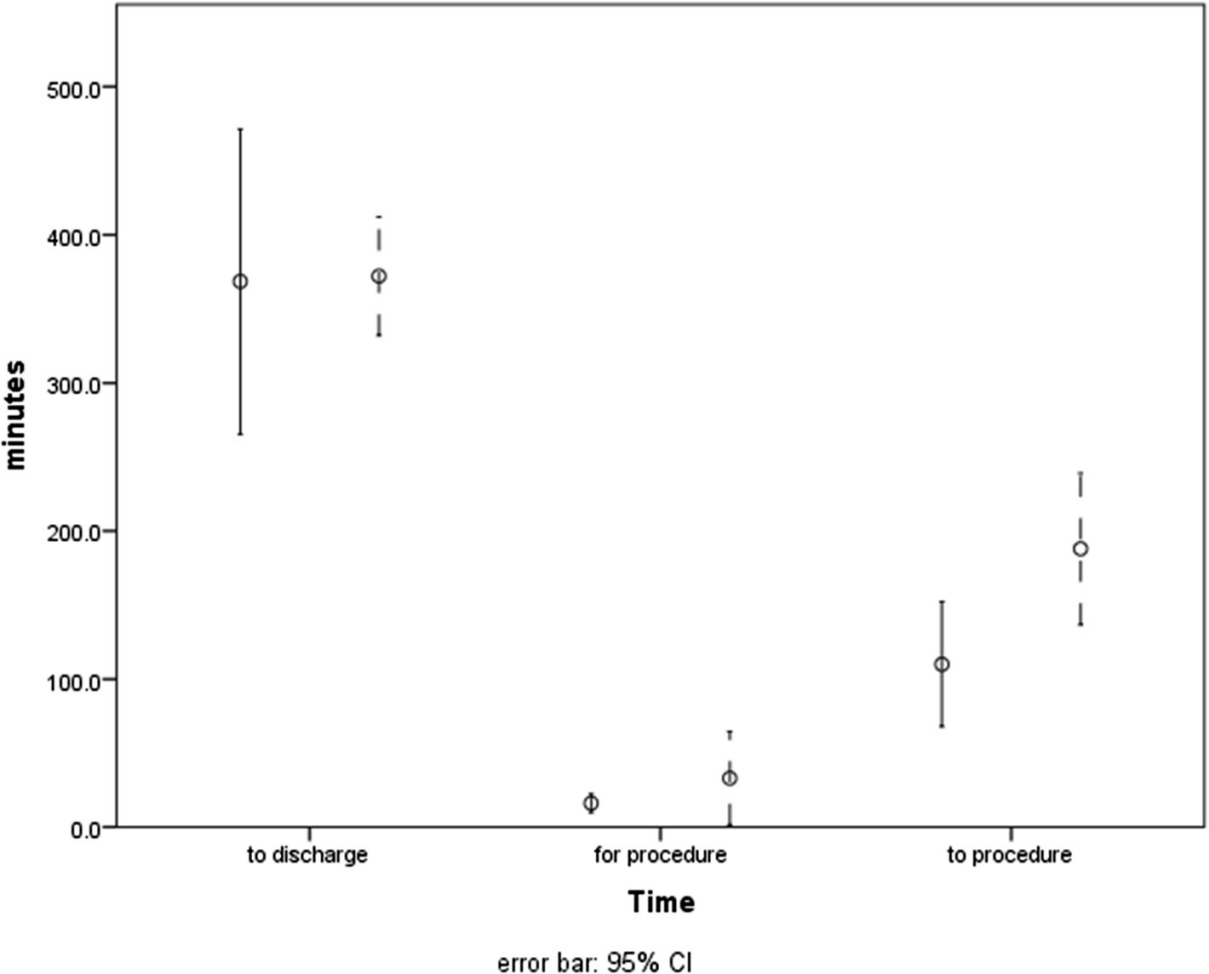
Clinical outcome evaluation of the treatment of luxations of the shoulder. Solid line: treatment by the emergency department team. Dotted line: treatment by the department of anesthesiology

The most common reason for sedation in the ED in 2015 was a luxated shoulder (*n* = 19). In comparison to 2014, when all sedations in the ED were conducted by the department of anaesthesiology, patients requiring sedation for luxated shoulders were of equal age and had comparable comorbidities as assessed by American Association of Anaesthesiologists Scale (ASA scale) (Table 3). While we did not find significant differences between patients treated in the ED and those treated by the Department of anaesthesiology regarding the patient’s time to discharge or the time required for the procedure, the time to the procedure was significantly faster in patients sedated by the ED team (*p* = 0.002; see Table 3 and Fig. 3, *d* = 0.88).

**Table 3:** Comparison of the treatment of shoulder luxations from 2014 (Dept. of Anaesthesia) vs. 2015 (ED)

	ED (*n* = 19)	Anesthesiology (*n* = 14)	*P*-value
Age	45.11 (23.68)	42.93 (20.02)	0.783
Gender (n female; %)	8 (42.1 %)	2 (14.3 %)	0.131
ASA (n, %)			
1	12 (63.16 %)	6 (42.86 %)	0.097
2	3 (15.78 %)	7 (50.00 %)	
3	4 (21.05 %)	1 (7.14 %)	
Time			
to procedure	111.05 (87.21)	187.93 (88.59)	0.002*
for procedure	16.11 (12.92)	33.00 (54.72)	0.942

*ED* emergency department, *ASA* American Association of Anaesthesiologists, mean (standard deviation), *: *p* < 0.05

## Discussion

Our study demonstrates, that interprofessional and interdisciplinary simulation-based sedation training can help to implement a sedation concept in an emergency department. We found that our training greatly increases the self-efficacy of participants, self-assessed knowledge about emergency procedures as well as knowledge about CRM-guidelines, regardless of professional experience or profession. Clinical outcome evaluation confirmed the concepts safety and feasibility.

Although participants had different professions and professional experience, the intervention uniformly improved their self-efficacy in all subgroups. This is important because interprofessional education requires careful consideration of individual backgrounds in advance if it is to be beneficial for everyone and to prevent one group from benefitting much more than another. A reason for this might be our focus on CRM, emergency procedures and team function, all of which are less dependent on previous knowledge and profession than educational activities that target more cognitive outcomes. The most important goal in interprofessional education is to foster team skills [25]. Effective communication within health care teams is among the most important factors in preventing medical error and collaboration among team member has previously been demonstrated to strongly affect patient outcomes [26–28]. Thus, any educational intervention targeted towards improved team collaboration will likely benefit patients.

Key factors in sedation procedures and the key to gaining confidence are the increase of knowledge of emergency procedures as well as knowledge of the applicable medication. The gains in knowledge of both emergency procedures and knowledge of sedation medication might further contribute to the increased self-efficacy. A perceived superiority of simulation-based team training of sedation procedures over lecture-only formats has also been found in previous studies [12].

The step-by-step checklist we provided was not only valued by participants, but it was also the impression of instructors and participants that it substantially improved the performance in all scenarios. This is consistent with the benefit of checklists and team timeouts in operating theatres, as publicised in the WHO surgical safety checklist [29, 30]. The effectiveness of checklists has been well evaluated in many environments [31–33]. Especially in emergency departments, a structured teaching approach in combination with a step-by-step checklist seems to be a valuable tool. However, the checklist used was not specifically evaluated in this study and requires further research.

Apart from the improvement in clinical and technical skills, the second field of knowledge in the training was related to human factors. Nearly half of the participants stated that they were completely unfamiliar with CRM principles before the training course but now intend to use CRM-principles on a regular basis. The principles of “calling for help early” and “ensuring effective communication”, were found to be most memorable to the participants in our study, and are in accordance with the idea of preventing critical situations, which we emphasised in our training.

Sometimes calling for help in emergency situations is complicated by either a culture of blame or the lack of support from senior staff members. Therefore we focussed on generating a culture of respect and support, but also on giving the participants clear guidelines, emergency routines and criteria for emergency procedures. Such clear criteria for calling for help are known to facilitate distress calls and emergency procedures [34]. The connection between team culture and performance has long been recognised [35].

### Clinical outcome evaluation

It has been previously suggested that increased self-efficacy is not necessarily linked to better objective performance, and this has been discussed in the literature [36, 37]. In contrast to this, self-efficacy becomes a self-fulfilling prophecy by actually increasing the chances of success in a given task [38]: humans are generally better in tasks we feel proficient in. In accordance with this conception of self-efficacy, Schubert et al. found that good performance was related to high levels of self-reported feelings of preparedness [39].

The limited number of procedures performed during the first year after implementation emphasises the need of a structured training as well as the need of regular refresher trainings. It is reassuring that all complications encountered during the real-life sedations could be mastered by the trained ED staff without harm to the patient. In patients with a luxated shoulder, the time to procedure in sedations done by the ED staff was shorter than the time to procedure when a anaesthesia team was needed. This is an important step on the way to minimize the time to reduction in order to protect patients from secondary damage. It remains unclear, why the time to discharge was not significantly different. A significant reduction in time to discharge could contribute to an improved patient flow in a ED and further improve patient satisfaction.

### Limitations

One limitation concerns the fact that we only measured self-efficacy and self-accessed knowledge after the training day and did not test objectively acquired knowledge. We instead included real-life outcome investigation, as our training, similar to other similar programs, aims to focus on team-work to improve patient outcome in contrast to classic teaching forms like class-room teaching [12]. Another possibility of outcome assessment would have been the video-assisted observation of actual performance, using objective behavioural marker systems. Unfortunately, this was not possible in our setting, because assessment including video-recordings is extremely resource demanding and sensitive regarding privacy. We would however argue that patient outcome is the clinically more relevant measure.

Second, the sample size of patients in this study is small. This further emphasises the need for standardised procedures and structured and repetitive training, since the personal experience of staff is likely limited as a consequence.

## Conclusions

In summary, we have developed an interprofessional and interdisciplinary simulation-based sedation training approach as an efficient and secure way to implement a sedation concept in an emergency department. Clinical outcome evaluation confirmed the concepts safety and feasibility. Furthermore, the concept is equally effective in raising self-efficacy in all participating professions.

## References

[1] Agrawal D, Manzi SF, Gupta R, Krauss B (2003). Preprocedural fasting state and adverse events in children undergoing procedural sedation and analgesia in a pediatric emergency department. Ann Emerg Med..

[2] Mallory MD, Baxter AL, Yanosky DJ, Cravero JP (2011). Emergency physician–administered propofol sedation: a report on 25,433 sedations from the pediatric sedation research consortium. Ann Emerg Med..

[3] Cravero JP, Beach ML, Blike GT, Gallagher SM, Hertzog JH (2006). Incidence and nature of adverse events during pediatric sedation/anesthesia for procedures outside the operating room: report from the pediatric sedation research consortium. Pediatrics..

[4] Weaver CS, Hauter WE, Brizendine EJ, Cordell WH (2007). Emergency department procedural sedation with propofol: is it safe?. J Emerg Med..

[5] Kuypers MI, Mencl F, Verhagen MF, Kok MF, Dijksman LM, Simons MP (2011). Safety and efficacy of procedural sedation with propofol in a country with a young emergency medicine training program. Eur J Emerg Med..

[6] Kohn LT, Corrigan JM, Donaldson MS (1999). To err is human: building a safer health system. The Institute of Medicine report on medical error. The Institute of Medicine.

[7] Mellin-Olsen J, Staender S, Whitaker DK, Smith AF (2010). The Helsinki declaration on patient safety in anaesthesiology. Eur J Anaesthesiol..

[8] Haynes AB, Weiser TG, Berry WR, Lipsitz SR, Breizat AH, Dellinger EP, Herbosa T, Joseph S, Kibatala PL, Lapitan MC, Merry AF, Moorthy K, Reznick RK, Taylor B, Gawande AA, Safe Surgery Saves Lives Study Group (2009). A surgical safety checklist to reduce morbidity and mortality in a global population. N Engl J Med.

[9] Winters BD, Gurses AP, Lehmann H, Sexton JB, Rampersad CJ, Pronovost PJ (2009). Clinical review: checklists - translating evidence into practice. Crit Care..

[10] World Health Organization. Framework for Action on Interprofessional Education and Collaborative Practice. Available at: http://www.who.int/hrh/resources/framework_action/en/. Accessed: March26, 2015.

[11] Wilbur L (2014). Interprofessional education and collaboration: a call to action for emergency medicine. Acad Emerg Med..

[12] Tobin CD, Clark CA, McEvoy MD, Reves JG, Schaefer JJ, Wolf BJ, Reeves ST (2013). An approach to moderate sedation simulation training. Simul Healthc..

[13] Kobayashi L, Dunbar-Viveiros JA, Devine J, Jones MS, Overly FL, Gosbee JW, Jay GD (2012). Pilot-phase findings from high-fidelity in Situ medical simulation investigation of emergency department procedural sedation. Simul Healthc..

[14] Smith AF (2007). Patient safety: people, systems and techniques. Acta Anaesthesiol Scand.

[15] Godwin SA, Burton JH, Gerardo CJ, Hatten BW, Mace SE, Silvers SM, Fesmire FM (2014). American College of Emergency Physicians. Clinical policy: procedural sedation and analgesia in the emergency department. Ann Emerg Med.

[16] Miner JR, Gray RO, Bahr J, Patel R, McGill JW (2010). Randomized clinical trial of propofol versus ketamine for procedural sedation in the emergency department. Acad Emerg Med..

[17] Hohl CM, Sadatsafavi M, Nosyk B, Anis AH (2008). Safety and clinical effectiveness of midazolam versus propofol for procedural sedation in the emergency department: a systematic review. Acad Emerg Med..

[18] Havel CJ, Strait RT, Hennes H (1999). A clinical trial of propofol vs midazolam for procedural sedation in a pediatric emergency department. Acad Emerg Med..

[19] American Society of Anesthesiologists Task Force on Sedation and Analgesia by Non-Anesthesiologists (2002). Practice guidelines for sedation and analgesia by non-anesthesiologists. Anesthesiology.

[20] Waugh JB, Epps CA, Khodneva YA (2011). Capnography enhances surveillance of respiratory events during procedural sedation: a meta-analysis. J Clin Anesth..

[21] Gaba DM, Howard SK, Fish KJ, Smith BE, Sowb YA (2001). Simulation based training in Anesthesia Crisis Resource Management (ACRM): a decade of experience. Simul. Gaming.

[22] Gaba DM, Fish KJ, Howard SK (1994). Crisis Management in Anesthesiology.

[23] Carne B, Kennedy M, Gray T (2012). Review article: Crisis resource management in emergency medicine. Emerg Med Australas..

[24] Rall M, Dieckmann P (2005). Safety culture and crisis resource management in airway management: general principles to enhance patient safety in critical airway situations. Best Pract. Res. Clin. Anaesthesiol..

[25] Thistlethwaite J (2012). Interprofessional education: a review of context, learning and the research agenda. Med Educ..

[26] Lingard L, Espin S, Whyte S, Regehr G, Baker GR, Reznick R, Bohnen J, Orser B, Doran D, Grober E (2004). Communication failures in the operating room: an observational classification of recurrent types and effects. Qual Saf Health Care.

[27] Sutcliffe KM, Lewton E, Rosenthal MM (2004). Communication failures: an insidious contributor to medical mishaps. Acad Med.

[28] Hauz WE, Kämmer J, Schauber SK, Spies CD, Gaissmaier W (2015). Diagnostic performance by medical students working individually or in teams. JAMA.

[29] Rodrigo-Rincon I, Martin-Vizcaino MP, Tirapu-Leon B, Zabalza-Lopez P, Zaballos-Barcala N, Villalgordo-Ortin P, Abad-Vicente FJ, Gost-Garde J (2015). The effects of surgical checklists on morbidity and mortality: a pre- and post-intervention study. Acta Anaesthesiol Scand..

[30] World Health Organization Guidelines for Safe Surgery. 2009. Available from http://whqlibdoc.who.int/publications/2009/9789241598552_eng.pdf. Accessed: February 20, 2016.

[31] Keroack MA, Youngberg BJ, Cerese JL, Krsek C, Prellwitz LW, Trevelyan EW (2007). Organizational factors associated with high performance in quality and safety in academic medical centers. Acad. Med..

[32] Pinsky HM, Taichman RS, Sarment DP (2010). Adaptation of airline crew resource management principles to dentistry. J. Am. Dent. Assoc..

[33] Eisen LA, Savel RH (2009). What went right: lessons for the intensivist from the crew of US Airways Flight 1549. Chest.

[34] Brindley PG (2010). Patient safety and acute care medicine: lessons for the future, insights from the past. Crit. Care.

[35] Krimsky WS, Mroz IB, McIlwaine JK (2009). A model for increasing patient safety in the intensive care unit: increasing the implementation rates of proven safety measures. Qual. Saf. Health Care.

[36] Barnsley L, Lyon PM, Ralston SJ, Hibbert EJ, Cunningham I, Gordon FC, Field MJ (2004). Clinical skills in junior medical officers: a comparison of self-reported confidence and observed competence. Med Educ.

[37] Duns G, Weiland T, Crotty B, Jolly B, Cuddihy H, Dent A (2008). Self-rated preparedness of Australian prevocational hospital doctors for emergencies. Emerg Med Australas.

[38] Eva K, Regehr G (2005). Self-assessment in the health professions: a reformulation and research agenda. Acad Med.

[39] Schubert A, Tetzlaff JE, Tan M, Ryckmann V, Mascha E (1999). Consistency, inter-rater reliability, and validity of 441 consecutive mock oral examinations in anesthesiology. Anesthesiology.

